# Phytochemical, Cytotoxicity, Antioxidant and Anti-Inflammatory Effects of *Psilocybe Natalensis* Magic Mushroom

**DOI:** 10.3390/plants9091127

**Published:** 2020-08-31

**Authors:** Sanah M. Nkadimeng, Alice Nabatanzi, Christiaan M.L. Steinmann, Jacobus N. Eloff

**Affiliations:** 1 Phytomedicine Programme, Paraclinical Sciences Department, University of Pretoria, P/Bag X04, Onderstepoort, Pretoria, Gauteng 0110, South Africa; alice2nabatanzi@gmail.com (A.N.); kobus.eloff@up.ac.za (J.N.E.); 2 Future Africa, University of Pretoria, Hatfield, Pretoria, Gauteng 0186, South Africa; 3 Department of Plant Sciences, Microbiology and Biotechnology, College of Natural Sciences, Makerere University, Kampala 00256, Uganda; 4 Physiology Department, Sefako Makgatho Health Sciences University, Ga-Rankuwa, Gauteng 0208, South Africa; chris.steinmann@smu.ac.za

**Keywords:** depression, antioxidant, cytokines, anti-inflammatory, cytotoxicity, medicinal mushroom, *Psilocybe natalensis*

## Abstract

Psilocybin-containing mushrooms, commonly known as magic mushrooms, have been used since ancient and recent times for depression and to improve quality of life. However, their anti-inflammatory properties are not known. The study aims at investing cytotoxicity; antioxidant; and, for the first time, anti-inflammatory effects of *Psilocybe natalensis*, a psilocybin-containing mushroom that grows in South Africa, on lipopolysaccharide-induced RAW 264.7 macrophages. Macrophage cells were stimulated with lipopolysaccharide and treated with different concentrations of *Psilocybe natalensis* mushroom extracted with boiling hot water, cold water and ethanol over 24 h. Quercetin and N-nitro-L-arginine methyl ester were used as positive controls. Effects of extracts on the lipopolysaccharide-induced nitric oxide, prostaglandin E_2_, and cytokine activities were investigated. Phytochemical analysis, and the antioxidant and cytotoxicity of extracts, were determined. Results showed that the three extracts inhibited the lipopolysaccharide-induced nitric oxide, prostaglandin E_2_, and interleukin 1β cytokine production significantly in a dose-dependent manner close to that of the positive controls. A study proposed that ethanol and water extracts of *Psilocybe natalensis* mushroom were safe at concentrations used, and have antioxidant and anti-inflammatory effects. Phytochemical analysis confirmed the presence of natural antioxidant and anti-inflammatory compounds in the mushroom extracts.

## 1. Introduction

Studies have shown an association between pathological inflammation and various chronic diseases, such as cancer and cardiovascular diseases [1]. Inflammation is known as a normal protective response to injury and/or infection. It involves complex processes that limit tissue injury in normal circumstances; however, in chronic inflammation, immune cells are dysregulated and tend to lose their self-limiting nature as a result thereof [1]. 

A number of inflammatory cytokines are involved in the pathogenesis of chronic inflammation [2]. Inflammatory cytokines are cell-signalling protein molecules that are released during inflammation and introduce signalling cascades that are able to activate the immune system [3]. Type 1 cytokines include tumour necrosis factor (TNF-α), and interferon-y and interleukin (IL) 1β, which play a primary role in enhancing the cellular immune response. Type 2, on the other hand, includes IL6, IL10 and IL13, which are more linked to antibody responses [4]. These cytokine also set in motion the activation of acute-phase proteins like C-reactive protein that further activate the immune system to release more cytokines and sustain the pathological inflammation state [4]. As a result, there is a higher uncontrolled release of pro-inflammatory cytokine such as TNF-α and IL 1β observed in many chronic diseases associated with pathological inflammation [2]. Oxidative stress also plays a significant role in the pathophysiology of inflammation via the actions of free radicals, non-radical molecules, and reactive oxygen and nitrogen species [5]. Lowered antioxidant concentrations and increased oxidative stress are also associated with mitochondrial dysfunctions and cell death [2]. In macrophages, lipopolysaccharide (LPS), a well-known endotoxin, induces productions of inflammatory mediators like inducible nitric oxide (iNO) and prostaglandinE_2_, which are synthesised by inducible nitric oxide synthase (iNOS) and cyclooxygenase-2, also known as prostaglandin endoperoxide sythase-2 (PGE_2_), respectively, in addition to pro-inflammatory cytokine activation [2,6]. As a result, LPS is commonly used as a potent inflammatory agent in different experimental models of inflammation studies [7]. 

Psilocybin-containing mushrooms, commonly known as magic mushrooms, are reported to have been used for centuries for their sacred, healing and mind-manifestation hallucinogenic powers within various indigenous societies [8,9]. Fatal intoxications due to exposure to magic mushrooms are rare and often reported to be mainly in combination with other drugs [10]. Furthermore, the lethal dose of magic mushrooms in rats is 280 mg/kg, and for humans is 17 kg/70 kg, which is very low, and magic mushrooms are therefore not normally considered toxic [10]. 

The crude water and sometimes ethanol extracts of psilocybin-containing mushrooms are the main source and method of treatment taken by many people. Many species of mushrooms are known to contain various molecules and scavenger-free radicals such as polysaccharides and phenol compounds [11]. Consequently, many naturally occurring substances in plants and mushrooms are perceived to possess antioxidant activities [11]. Studies have been performed on magic mushrooms with regards to their antidepressant properties; however, very little is known of their antioxidant potential and, to the best of our knowledge, there are no scientific reports on their anti-inflammatory potentials or properties. 

This study aimed at investigating the cytotoxicity; antioxidant; and, for the first time, anti-inflammatory effects of *Psilocybe natalensis* mushroom, commonly known as “Natal Super strength”, which is one of the well-known psilocybin mushrooms in the family Strophariaceae and genera of *Psilocybe*, that grow in South Africa on LPS-induced RAW264.7 macrophages [9]. The *Psilocybe natalensis* mushroom spores were identified and authenticated with an SKU number NSS-1 by the Spore Spot Company, Durban, South Africa. We hypothesize that the water and ethanol extracts of *Psilocybe natalensis* mushroom have anti-inflammatory properties. These results will reveal for the first time the potential anti-inflammatory mechanism offered by *Psilocybe natalensis* magic mushroom. 

## 2. Results

### 2.1. ABTS Free Radical Scavenging Activity of P. natalensis Extracts 

The extracts and positive controls, trolox and ascorbic acid, had concentration-dependent effect on the antioxidant activity. The positive controls showed inhibitory concentration (IC_50_) very low under 1 µg/mL, as shown in Table 1. The ethanol displayed an IC_50_ less than 50 µg/mL, while the hot water and cold water showed IC_50_ greater than 50 µg/mL but less than 100 µg/mL.

### 2.2. Cytotoxicity of P. natalensis Extracts 

The lethal concentrations (LC_50_) of the extracts were higher than the positive control—doxorubicin—on measurements of toxicity on normal Vero cells, as shown on Table 1. The ethanol extracts were mostly safe on the viability of cells with LC_50_ > 100 µg/mL, followed by hot water and cold water, which had the lowest LC_50_ of 25 µg/mL, Table 1.

### 2.3. Anti-Inflammatory Effects of the Extracts 

#### 2.3.1. Inhibitory Effects of *P. natalensis* Extracts on Inducible NO Production and % Cell Viability 

**Figure 1:**
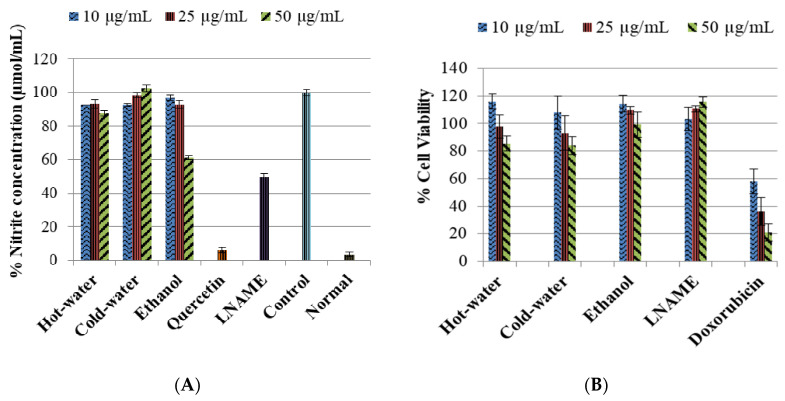
The inhibitory effect of *P. natalensis* extracts on LPS-induced NO production (**A**) and % cell viability (**B**) on RAW 264.7 macrophages treated with different concentrations (10, 25 and 50 µg/mL) and positive controls; for Figure 1A: quercetin (50 µg/mL), N-Nitro-L-Arginine Methyl Ester (LNAME) (100 µM) and for Figure 1B: LNAME (25, 50 and 100 µM) and doxorubicin (4, 10 and 20 µM) over 24 h.

The control LPS-induced cells significantly increase (*p* < 0.001) the nitrite content compared to the normal cells, as shown in Figure 1. The positive controls—quercetin and LNAME—both inhibited the inducible NO significantly (*p* < 0.001 and *p* < 0.001, respectively) compared to the control cells. Figure 1 also shows the hot-water extract significantly decreased the inducible NO with all the concentrations 50 µg/mL (*p* < 0.001), 25 µg/mL (*p* < 0.001) and 10 µg/mL (*p* < 0.001) in comparison to the control cells. The ethanol extract significantly decreased the iNO with the 50 µg/mL (*p* < 0.001) very close to LNAME values and 25 µg/mL (*p* < 0.001) but non-significantly with 10 µg/mL (*p* = 0.299) compared to the control cells. Meanwhile the cold-water significantly inhibited the iNO only with the lowest concentration 10 µg/mL (*p* < 0.001). The results also showed that the extracts were not toxic to the macrophage cells in comparison to doxorubicin, the toxic positive control, Figure 1. Furthermore, the % cell viability of treated cells where iNO production was significantly inhibited were safe at ≥80% cell viability. 

#### 2.3.2. Effects of *P. natalensis* Extracts on PGE_2_ Production 

**Figure 2:**
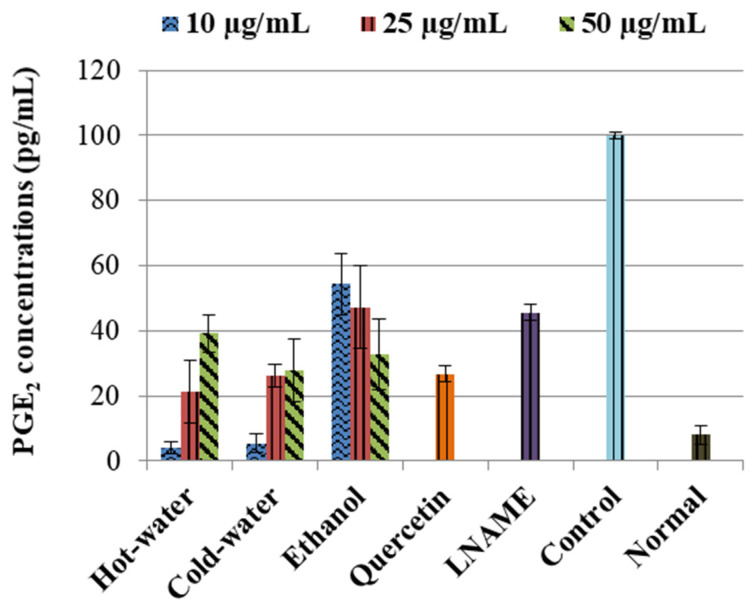
Inhibitory effects *of P. natalensis* extracts on LPS-induced PGE_2_ production on RAW 264.7 macrophages treated with different concentrations (10, 25 and 50 µg/mL) and positive controls; quercetin (50 µg/mL) and LNAME (100 µM) over 24 h.

The control LPS-induced cells significantly (*p* < 0.001) increased the concentration of PGE_2_ in comparison to the normal cells, as shown in Figure 2. The positive controls—quercetin and LNAME—significantly reversed and inhibited the PGE_2_ concentrations *p* < 0.001 and *p* < 0.001 respectively compared to the control cells. The three extracts also significantly inhibited the PGE_2_ concentrations in comparison to the control cells, see Figure 2. The ethanol had an accelerating dose-dependent inhibition that increased as concentration increased, and potent inhibition was with 50 µg/mL (*p* < 0.001) concentration, which was very close to quercetin and lower than LNAME. The hot-water and cold-water, on the other hand, had a deceleration dose-dependent response where PGE_2_ inhibition was weakened as concentrations increases. The potent inhibition for both hot-water and cold-water was with the lowest concentration 10 µg/mL (*p* < 0.001 and *p* < 0.001, respectively), which was lower than both LNAME and quercetin and very close to the normal cells.

#### 2.3.3. Effects of *P. natalensis* Extracts on Cytokine Production

**Figure 3:**
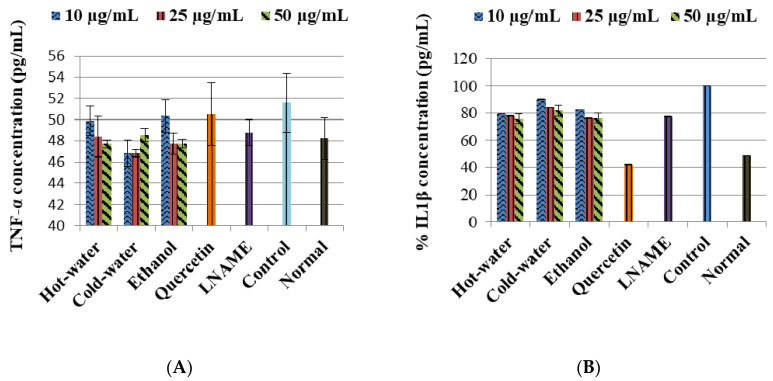
Effects of *P. natalensis* extracts on LPS-induced TNF-α (**A**) and IL1β (**B**) production in RAW 264.7 macrophages treated with different concentrations (10, 25 and 50 µg/mL), and positive controls quercetin (50 µg/mL) and LNAME (100 µM) over 24 h.

**Figure 4:**
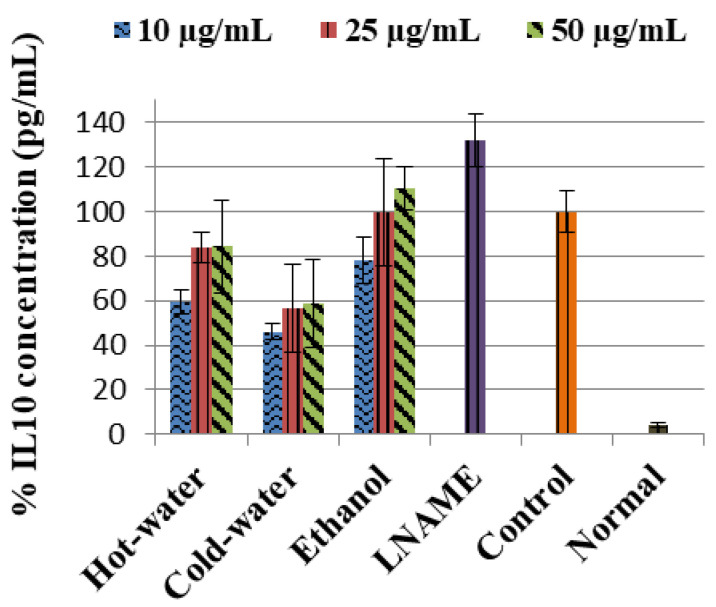
Effects of *P natalensis* extracts on LPS-induced IL10 production in RAW 264.7 macrophages treated with different concentrations (10, 25 and 50 µg/mL) and positive control LNAME (100 µM) over 24 h.

The LPS-induced control cells increased the TNF-α non-significantly while IL1β (*p* < 0.001) and IL10 (*p* < 0.001) were significantly increased in comparison to the normal cells (Figure 3). Moreover, the three extracts significantly decreased the IL1β production very closed to LNAME. The ethanol extract significantly increased the IL10 production with 50 µg/mL (*p* < 0.001) same as LNAME (*p* < 0.001) in comparison to the control, while the hot-water and cold-water extracts decreased it in a dose-dependent response but were still higher than normal non-induced cells, Figure 4. 

#### 2.3.4. Phytochemical Determination

**Figure 5:**
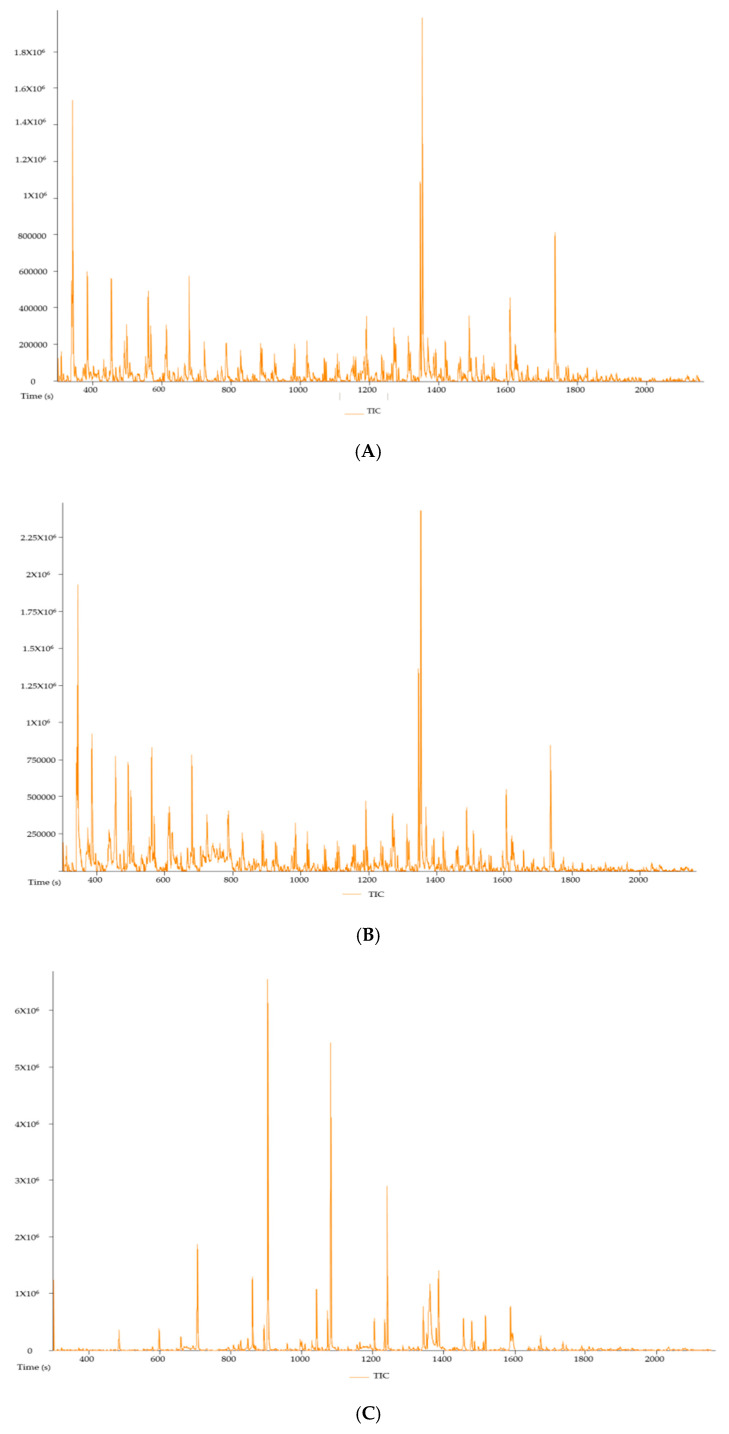
Gas chromatography-mass spectrometry (GCMS-MS) chromatogram of hot-water (**A**), cold-water (**B**) and 70% ethanol (**C**) extracts of *P. natalensis* mushroom.

Figure 5 shows the gas chromatography-mass spectrometry (GCMS-MS) chromatograms of the hot-water, cold-water and ethanol extracts of *P. natalensis* mushrooms, respectively. The compounds with known antioxidant and anti-inflammatory from the chromatograms of the three extracts are tabulated in Table 2 with their molecular weight, formulas and area % per extracts. There were six known compounds with natural antioxidant and anti-inflammatory activities extracted from the three *P. natalensis* mushroom extracts. Compound n-hexadecoid acid with known antioxidant and anti-inflammatory activity was found to be present in all the three extracts with different degrees of area percentage. Ethanol extracts had the highest area % of compounds like nonadecane and tetradecane, which are known to possess anti-inflammatory and antioxidant properties along with other medicinal benefits that were not derived from the water extracts. 

## 3. Discussion

The antioxidant potential of the extracts was determined using ABTS assay, which is one of the most commonly used radical scavenging assays methods for evaluating different natural product and functional food materials because of its quick, reproducible and inexpensive properties [19]. Phongpaichit et al. [20] state that the radical scavenging activity of extracts with IC_50_ < 100 µg/mL has good antioxidant potential while the ones with IC_50_ < 50 μg/mL are considered potent antioxidant agents. Accordingly, the hot-water and cold-water extracts of *P. natalensis* mushroom had good antioxidant potential while the ethanol had potent activity. Since increased oxidative stress and lowered antioxidant concentrations play a great role in the genesis and progression of inflammation, the scavenging properties of *P. natalensis* mushroom extracts will be of beneficial in chronic inflammation treatment [2]. Cytotoxicity results showed that the extracts were safe on normal Vero cells compared to positive control doxorubicin. Moreover, the results also indicated that the ethanol and water extracts of *P. natalensis* mushroom will be regarded as safe in relation to the American National Cancer Institution guidelines, which state that an extract with LC_50_ ≤ 20 μg/mL is toxic [21]. 

Macrophage cells are reported as key immune cells in the initial stage of inflammation [22]. According to [23], RAW 264.7 macrophage cells provide an excellent model in drug screening for their potential use in inflammatory diseases and to evaluate potential inhibitors of pathways leading to inducible NO production. As a result, when the RAW cells are induced with LPS, they lead to a series of responses that include synthesis and production of prostanoids and pro-inflammatory cytokines present in pathological inflammation illnesses. Our study showed that the control LPS-induced cells significantly increase in iNO, PGE_2_, IL1β and IL10 productions and TNF-α non-significantly in comparison to the normal cells. This was in agreement with other studies confirming the activation of signalling pathways, which leads to the release of pro-inflammatory cytokines, including IL1 β, TNF-α and mediators like NO and PGE_2_ and anti-inflammatory cytokines like IL10 induced by LPS in RAW macrophage cells in the study [24,25,26]. The positive controls LNAME and quercetin in our study significantly reversed these effects. 

The three extracts of *P. natalensis* magic mushroom dose-dependently inhibited iNO production, and the effects were more pronounced with the highest concentration of the ethanol extract. Furthermore, these inhibition effects were not due to cytotoxicity, as shown in Figure 1, where the percentage cell viability of the extracts was ≥80% in safe margins in all the concentrations where significant iNO inhibition was observed. Since iNO accumulation is a major macrophage-derived inflammatory mediator involved in the pathogenesis of various inflammation diseases, inhibiting or controlling its production is significant in anti-inflammatory investigation [25,27]. By suppressing iNO production, the extracts demonstrated an important healing potential effect in pathological inflammation. 

The three extracts inhibited the LPS-induced PGE_2_ production, which is another mediator of inflammation significantly close to the LNAME and quercetin. During inflammation, induced increase in PGE_2_ production is also associated with the occurrence of pathological pain; as a result, the inhibitory properties of *P. natalensis* mushrooms extracts on PGE_2_ demonstrated their potential ability to alleviate inflammation and physical chronic pains associated with pathological inflammation [28,29]. 

With regards to the cytokine activities, the hot-water, cold-water and ethanol extracts of *P. natalensis* mushroom decreased significantly the LPS-induced pro-inflammatory cytokines IL1β and reduced TNF-α non-significantly. Only the ethanol extract at the highest concentration used significantly increased the anti-inflammatory cytokine IL10 above the control in line with LNAME, while the other extracts down-regulated it in a dose-dependent manner. It is known that high levels of pro-inflammation cytokines like IL1β and TNF-α are present in the pathogenesis of many chronic diseases associated with chronic inflammation, and that they contribute to cell injury and damage and also initiate or sustain the inflammation state [4,30]. By suppressing the LPS-induced production of IL1β and TNF-α cytokines, the *P. natalensis* mushroom extracts demonstrate another positive potential benefit in chronic inflammation treatment. 

These *P. natalensis* mushroom extracts effects were confirmed by the GCMS-MS analysis, which showed that the three mushroom extracts contained compounds known to induce natural antioxidant and anti-inflammatory effects such as n-hexadecanoic acid; 4h-Pyran-4-one, 2,3-dihydro-3,5-dihydroxy-6-methyl-; 3-octanone; and dibutyl phthalate. The GCMS-MS results also showed that the ethanol extract had very high percentage area of compounds known to have antioxidant and anti-inflammatory effects such as nonadecane and tetradecane, as shown in Table 2. As a result, the phytochemical analysis supported the greater positive effects that were observed with the ethanol extracts in comparison to the water extracts in the study. 

In summary, these results indicated that the water and ethanol extracts of *Psilocybe natalensis* mushroom have potential antioxidant activity, an important factor in oxidative stress dysfunctions associated with inflammation. The results also indicated that the extracts will be considered safe in concentrations used. The study showed that the three extracts inhibited induced NO and PGE_2_ productions, the two inflammatory mediators involved in pathogenesis of inflammatory diseases. Furthermore, the results proposed that the hot-water, cold-water and ethanol extract lowered LPS-induced TNF-α and inhibited induced pro-inflammatory cytokine IL1β, the well-known pro-inflammatory cytokines reported to be high in chronic inflammations, and their reduction was associated with improvement in the diseases [31]. Moreover, the ethanol extract increased the concentrations of anti-inflammatory cytokine IL10. The GCMS-MS analysis supported these findings by showing presence of compounds known to induce natural antioxidant and anti-inflammatory compounds in the three extracts.

## 4. Materials and Methods 

### 4.1. Ethical Clearances

The protocol for this study was approved by University of Pretoria Research Ethics Committee, and the protocol number REC045-18 was assigned. Since psilocybin mushrooms are schedule 7 substances in South Africa, approval by the South African Department of Health Medical Control Council (MCC) was applied for, and a permit license POS 223/2019/2020 was given for the project. 

### 4.2. Mushroom Growth and Making Extracts 

The spore prints syringe of *Psilocybe natalensis* (*P. natalensis*) mushroom also known as “Natal super strength” were identified and authenticated with an SKU number NSS-1by the Spore Spot Company, Durban, South Africa, together with a growing sterile substrate kit (SSK-2), and were both purchased from the Spore Spot Company. Upon arrival, the spores were inoculated in a sterile substrate in sterile conditions in a triple locked laboratory under strict supervision as required by the MCC department. As soon as mycelium started to colonise about 60% of the substrate, it was transferred into a sterile monotub designed and donated by Mr L. Morland for the project. The temperature was controlled with an air conditioner and humidity supplied using a Clicks humidifier. As soon as the mushrooms fruit and mature, they were harvested and dried in an open oven at 35–36 °C over one to two days. The dried mushrooms were grounded into fine powder using a grinder. Extracts were made by measuring 5 g of dried powder into small sterile beaker and dissolved with 50 mL of boiling distilled water, cold water and 70% ethanol. The mixture was stirred using a stirrer for 5 min and allowed to stand for 24 h. After 24 h, the mixtures were filtered into small vials no 6 (Lasec, Johannesburg, South Africa) that were previously weighed and dried over night at 30 °C in an open oven. The extracts’ yield was calculated and stored in dark in a fridge until use. 

### 4.3. Free Radical Scavenging Activity on ABTS

The 2,2′-azinobis-(3-ethylbenzothiazoline-6-sulfonic acid) (ABTS) (Sigma-Aldrich, Johannesburg, South Africa) scavenging activity of the three extracts was assessed using methods of [32]. Ascorbic acid and trolox were used as positive controls. Briefly, freshly prepared ABTS was added into the two wells of each extract sample, while methanol was added into the other two wells to be used as blank, and the ability of the mushroom extracts to scavenge ABTS radicals was determined using a microplate reader (Biotek, Synergy HT, Analytical & Diagnostic Products CC, Johannesburg, South Africa) at a wavelength of 734 nm. The experiments were repeated three times, and inhibition concentration(IC) measured as IC_50_ values were calculated according to the formula: % ABTS inhibition = ((Absorbance control − Absorbance sample)/Absorbance control) × 100. The antioxidant ability was expressed as IC_50_ value, which is the concentration of the sample necessary to inhibit ABTS by 50%.

### 4.4. Cytotoxicity of Extracts on Vero Normal Cells

Viability of cells was determined using the tetrazolium-based colorimetric (MTT) assay described by [33] with modifications on normal African green monkey kidney (Vero) cells purchased from American Type Culture Collection (ATCC, Manassas, VA, USA) [34]. When cells had reached confluent culture, they were harvested and centrifuged at 200× *g* for 5 min and then resuspended in growth medium to 1 × 10^4^ cells/mL into each well of columns 2 to 12 of a sterile 96-well microtitre plate, and column 1 was used as blank (no cells). The growth medium used was Minimal Essential Medium (MEM), (PAN Biotech, Biocom Africa, Johannesburg South Africa) supplemented with 0.1% gentamicin (Virbac, Johannesburg, South Africa) and 5% foetal calf serum (separation scientific, Johannesburg, South Africa). The plates were incubated for 24 h at 37 °C in a 5% CO_2_ incubator. Then, MEM was aspirated from the cells and replaced with 200 µL of a serial prepared concentration range of extract. The serial dilutions of the test extracts were prepared in sera-free MEM. The microtitre plates were incubated at 37 °C in a 5% CO_2_ incubator for 48 h with extracts. Untreated cells (negative control) and positive control (doxorubicin chloride, Pfizer Laboratories, Johannesburg, South Africa) were included. 

After incubation, the media (with and without treatment) were aspirated, and cells were washed with 200 μL phosphate buffered saline (PBS) (Whitehead Scientific, Johannesburg, South Africa). Then, 100 µL of media was added to all the wells and 30 µL MTT (Inqaba biotec, Pretoria, South Africa, stock solution of 5 mg/mL in PBS) was added to each well and the plates incubated for a further 4 h at 37 °C. After incubation with MTT, the medium in each well was carefully removed without disturbing the MTT crystals in the wells. The MTT formazan crystals were dissolved by adding 50 µL DMSO to each well. The plates were shaken gently until the MTT solution was dissolved. The amount of MTT reduction was measured immediately by detecting absorbance in a microplate reader (Biotek, Synergy HT, Analytical & Diagnostic Products CC, Johannesburg, South Africa) at a wavelength of 540 nm and a reference wavelength of 630 nm. The wells in column 1, containing medium and MTT but no cells, were used to blank the plate reader. Viability of cells in percentages was calculated using the formula: % Viability = ((Sample Absorbance/control Absorbance) × 100). The experiments were performed in triplicate and repeated twice in different times. The lethal concentrations (LC_50_) values were calculated as the concentration of test extract resulting in a 50% reduction of absorbance compared to untreated cells. The 50% lethal concentration of the samples and positive control doxorubicin were obtained by linear regression analysis of concentration-response curve plotting between percentage of viability and sample concentration of two independent assays.

### 4.5. Anti-Inflammatory Effects of the Extracts 

#### 4.5.1. RAW 264.7 Macrophage Cell Culture

The RAW 264.7 macrophage cells purchased from American Type Culture Collection (ATCC, Manassas, VA, USA USA) were used in this study and maintained in Dulbecco’s modified Eagle’s medium (DMEM) (Pan Biotech, Separations scientific) supplemented with 10% Foetal bovine serum (Gibco, Sigma-Aldrich, Johannesburg, South Africa) and 1% of penicillin (100 units/mL) and streptomycin (100 μg/mL) (Celtic Molecular Diagnostics) at 37 °C in a 5% CO_2_ atmosphere (HERAcell 150, Thermo Electron Corp., Separation scientific, Johannesburg, South Africa). The cells were allowed to grow, and reached 80% confluence before being used in the experiments. 

#### 4.5.2. Cytotoxicity of Extracts on LPS-Induced RAW 264.7 Macrophages

The RAW 264.7 cells were seeded at a density of 4 × 10^4^ cells/well into each well of column 2 to 11 of sterile tissue culture treated 96 well plates (NEST, Whitehead scientific, Johannesburg, South Africa) and incubated for 24 h at 37 °C in a 5% CO_2_. Then, media was aspirated from all the wells and replaced with fresh medium. The cells were treated with lipopolysaccharide (LPS) (Sigma-Aldrich, Johannesburg, South Africa) at a concentration of 1 µg/mL in the presence of different concentrations of the three extracts (10, 25 and 50 µg/mL) and incubated for 24 h. Cytotoxicity was measured using MTT assay same as with the Vero cells above. Viability of cells in percentages was calculated using the formula: % Viability = ((Sample Absorbance/control Absorbance) × 100). The experiments were repeated two times on different occasions. 

#### 4.5.3. Treatment with the Extracts

The RAW 254.7 macrophage cells were plated 1 × 10^6^ cells per 25 cm^2^ tissue culture flasks (NEST, Whitehead scientific, Johannesburg, South Africa) over 24 h. Then, the medium was removed, fresh media added and the cells were stimulated with LPS 1 µg/mL and treated with the extracts at 10, 25 and 50 μg/mL concentrations. Quercetin (Sigma-Aldrich, Johannesburg, South Africa), a well-known antioxidant and a flavonol found in many fruits and plants, and N-Nitro-L-Arginine Methyl Ester (LNAME) (Sigma-Aldrich, Johannesburg, South Africa), a nitric oxide synthase (NOS) inhibitor, were used as positive controls. Control cells were stimulated with LPS but not treated. Normal cells were cells that were neither stimulated with LPS nor treated with extracts. The cells were exposed to LPS and treated over 24 h. After 24 h, medium was removed and stored in −80 freezer until day of analysis.

#### 4.5.4. Nitrite Content Measurements

To measure nitric oxide production in cell culture media after 24 h of treatments in LPS-induced macrophage cells, nitrite content was measured in cell culture supernatant as an indication of NO production based on the Griess reaction (Sigma-Aldrich, Johannesburg, South Africa). Briefly, 100 µL of cell culture supernatant was added to 100 µL of Griess reagent incubated for 15 min and absorbance measured at 540 nm. The concentration of nitrite was determined from the serial diluted standard curve.

#### 4.5.5. PGE_2_ Activity Measurements

The effects of the extracts on PGE_2_ were determined using the mouse PTGS_2_/COX-2 Prostaglandin endoperoxide synthase 2 (PGE_2_) ELISA kit (Elabscience, Biocom Africa, Johannesburg South Africa) according to the manufacture manual protocol. Concentrations of mouse PGE_2_ in the cell culture media samples were calculated from the standard curve. The absorbance was directly proportional to the concentrations of PGE_2_ in the sample medium.

#### 4.5.6. Cytokine Activity Measurements

The effects of the extracts on levels of TNF-α, IL1 β and IL10 were determined and quantified using the mouse ELISA kits with catalogue numbers E-EL-M0049, E-EL-M0037 and E-EL-M0046, (Elabscience, Biocom Africa, Johannesburg South Africa)), respectively, following the same protocol as above using the instructor manual on the cell culture medium. 

### 4.6. Phytochemical Determination of the Extracts

Phytochemical determination of extracts was performed using the gas chromatography-mass spectrometry (GCMS-MS) by the LC-MS (Synapt Waters, Johannesburg, South Africa) facility at the Chemistry Department, University of Pretoria. The water and ethanol extracts of *Psilocybe natalensis* mushrooms were dissolved in methanol (1 mg/mL). Chromatograms and presence of compounds in the three extracts were produced.

### 4.7. Statistical Analysis

Results are expressed as mean ± standard deviations, and statistically significant values were compared using one-way ANOVA analysis of variance using an interactive statistical program (Sigmastat, SPSS version 26, San Jose, CA, USA) and pairwise multiple comparison procedures using Holm—Sidak method. Normality testing was done using Shapiro—Wilk and equal variance test using Brown—Forsythe. The *p*-value of ≤0.050 was considered statistically significant. 

## 5. Conclusions

The results proposed for the first time that the water and ethanol extracts of *Psilocybe natalensis* are safe in the concentrations used, and have antioxidant and anti-inflammatory properties. The study recommends further investigations of the mushroom effects in vivo in this field. 

## Figures and Tables

**Table 1:** 2,2′-azinobis-(3-ethylbenzothiazoline-6-sulfonic acid) (ABTS) radical scavenging activity of the extracts and their cytotoxicity effect on Vero cells.

Sample	ABTS IC_50_ (μg/mL)	Vero LC_50_ (μg/mL)
Hot-water	86.233 ± 2.370	49.080 ± 3.340
Cold-water	90.154 ± 8.748	25.046 ± 0.460
70% Ethanol	26.586 ± 5.378	182.190 ± 11.860
Ascorbic acid	0.026 ± 0.003	not applicable
Trolox	0.928 ± 0.006	not applicable
Doxorubicin	not applicable	10.000 ± 1.327

**Table 2:** Compounds identified in the cold-water, hot-water and 70% ethanol extracts of *P. natalensis* mushroom with antioxidant and anti-inflammatory activities.

Compound Name	MW	Formula	Area %			Activity	Reference
			Cold	Hot	Ethanol		
n-Hexadecanoic acid	256	C_16_H_32_O_2_	1.7129	2.0765	2.313	Anti-inflammatory	[12]
						Antioxidant	[13]
4H-Pyran-4-one, 2,3-dihydro-	144	C_6_H_8_O_4_	2.0452			Antioxidant	[13]
3,5-dihydroxy-6-methyl-						Anti-inflammatory	
3-Octanone	128	C_8_H_16_O	3.6742	3.2977		Antioxidant	[14]
						Anti-inflammatory	
Dibutyl phthalate	278	C_16_H_22_O_4_		12.383		Anti-inflammatory	[15]
Nonadecane	268	C_19_H_40_			19.7244	Antioxidant	[16,17]
						Anti HIV	
						Antibacterial	
						Antimalarial	
Tetradecane	198	C_14_H_30_			17.1872	Anti-inflammatory	[18]
						Antimicrobial	
						Anti-diarrhoeal	

MW: Molecular weight.
